# Could Intravenous Immunoglobulin Collected from Recovered Coronavirus Patients Protect against COVID-19 and Strengthen the Immune System of New Patients?

**DOI:** 10.3390/ijms21072272

**Published:** 2020-03-25

**Authors:** Samir Jawhara

**Affiliations:** 1 CNRS, UMR 8576 - UGSF—Unité de Glycobiologie Structurale et Fonctionnelle, INSERM U1285, F-59000 Lille, France; samir.jawhara@univ-lille.fr; Tel.: +33-(0)3-20-62-35-46; Fax: +33-(0)3-20-62-34-16; 2 University of Lille, F-59000 Lille, France

**Keywords:** coronavirus, IVIg, immunotherapy, nCoV-2019, virus

## Abstract

The emergence of the novel coronavirus in Wuhan, China, which causes severe respiratory tract infections in humans (COVID-19), has become a global health concern. Most coronaviruses infect animals but can evolve into strains that cross the species barrier and infect humans. At the present, there is no single specific vaccine or efficient antiviral therapy against COVID-19. Recently, we showed that intravenous immunoglobulin (IVIg) treatment reduces inflammation of intestinal epithelial cells and eliminates overgrowth of the opportunistic human fungal pathogen *Candida albicans* in the murine gut. Immunotherapy with IVIg could be employed to neutralize COVID-19. However, the efficacy of IVIg would be better if the immune IgG antibodies were collected from patients who have recovered from COVID-19 in the same city, or the surrounding area, in order to increase the chance of neutralizing the virus. These immune IgG antibodies will be specific against COVID-19 by boosting the immune response in newly infected patients. Different procedures may be used to remove or inactivate any possible pathogens from the plasma of recovered coronavirus patient derived immune IgG, including solvent/detergent, 60 °C heat-treatment, and nanofiltration. Overall, immunotherapy with immune IgG antibodies combined with antiviral drugs may be an alternative treatment against COVID-19 until stronger options such as vaccines are available.

The emergence of the novel coronavirus in Wuhan, China, which causes severe respiratory tract infections in humans (COVID-19), has become a global health concern. Most coronaviruses infect animals but can evolve into strains that can also infect humans. Recently, we showed that intravenous immunoglobulin (IVIg) treatment reduces inflammation of intestinal epithelial cells and eliminates overgrowth of the opportunistic human fungal pathogen *Candida albicans* in the murine gut in association with downregulation of proinflammatory mediators combined with upregulation of anti-inflammatory cytokines [1].

Coronaviruses are enveloped positive-stranded RNA viruses belonging to the family Coronaviridae [2]. An envelope-anchored spike protein promotes coronavirus entry into host cells by first binding to a host receptor and then fusing viral and host membranes [2]. Whole-genome sequencing of viral RNA has revealed that the virus causing COVID-19 is phylogenetically related to the SARS-related coronaviruses first isolated in Chinese horseshoe bats during 2015‒2017 [3,4]. Researchers in Guangzhou, China, have recently suggested that pangolins are the probable animal source of the COVID-19 outbreak [5]. In terms of the interaction between the virus and its host, Lu et al. have reported that angiotensin-converting enzyme 2 (ACE 2) is most probably used by the spike protein of the COVID-19 virus as a receptor similar to that SARS-CoV [6].

Recently, Tang et al. showed that the COVID-19 has evolved into two major lineages—dubbed ‘L’ and ‘S’ types. The older ‘S-type’ appears to be milder and less infectious, while the ‘L-type’, which emerged later, spreads quickly and is currently more aggressive than the S-type [7]. Current symptoms reported for patients with COVID-19 have included mild to severe respiratory illness with fever, fatigue, cough, myalgia, and difficulty breathing [8]. Tyrrell et al. showed that infected respiratory epithelial cells by coronavirus become vacuolated and show damaged cilia that lead to production of inflammatory mediators, which increase nasal secretion and cause local inflammation and swelling [9]. These responses in turn stimulate sneezing, obstruct the airway, and raise the temperature of the mucosa [9].

Currently, there is no single specific vaccine or effective antiviral therapy against COVID-19. Several pharmaceutical and biotechnological companies are working on vaccine development and estimate that this vaccine will take years to develop and test before it can reach a large population. Additionally, there are currently no approved treatments for any coronavirus disease, including COVID-19. Several antiviral drugs are being tested, and initial findings are expected soon. Individuals with weakened immune systems appear to be at greater risk of developing complications associated with COVID-19. Immunotherapy using IgG in combination with antiviral drugs could be used to treat or prevent COVID-19 and to strengthen our immune response against this virus [10,11]. IgG antibodies include two functional portions: the F(ab′)_2_ fragment, which is responsible for antigen recognition, and the crystallizable fragment (Fc), which is important for activation of the immune response by interacting with Fcγ receptors on B-cells and other innate immune cells [12]. The Fc fragment also plays an important role in the activation of complement and in the clearance of microorganisms [12].

IVIg is a pool of IgG from thousands of healthy donors, and exposure of individual donors to endemic infectious diseases, vaccines, and ubiquitous microorganisms participates in the production of IgG antibodies against different microorganisms and their products [13,14,15].

IVIg has been used to treat patients with autoimmune and chronic inflammatory diseases, such as dermatomyositis, Kawasaki disease, multiple sclerosis, lupus, chronic lymphocytic leukemia, and idiopathic thrombocytopenic purpura [16,17,18]. Furthermore, IVIg has also been used as an anti-infectious agent against viruses, bacteria, and fungi in human patients and experimental models [13,19,20,21]. IVIg treatment may result in some adverse events, which are associated with specific immunoglobulin preparations and individual differences, but many clinical and experimental studies show that switching from IVIg to subcutaneous immunoglobulin can minimize these adverse events [22,23,24].

IVIg plays an important role in the prevention of infectious episodes in primary immunodeficient patients, and the beneficial effects of these antibodies in the treatment of infectious diseases goes beyond simple neutralization of microorganisms or their toxins. Anti-inflammatory pathways are also critical for protection against infection [25].

IVIg may modulate the immune response via multiple mechanisms, including blocking a wide array of proinflammatory cytokines, Fc-gamma receptors (FcγRs), and leukocyte adhesion molecules, suppressing pathogenic Th1 and Th17 subsets, and neutralizing pathogenic autoantibodies [26,27,28]. IVIg can also expand regulatory T-cells by induction of cyclo-oxygenase-2-dependent prostaglandin E2 production in dendritic cells [29].

In our study, IVIg treatment reduced intestinal inflammation and decreased *Escherichia coli*, *Enterococcus faecalis*, and *C. albicans* populations in the gut of mice [1]. Overgrowth of *E. coli* and *E. faecalis* populations is known to be involved in dysbiosis of the gut microbiota in inflammatory bowel diseases (IBDs), which are chronic inflammatory conditions of the gastrointestinal tract [30,31]. We also showed that the beneficial effects of IVIg were associated with suppression of inflammatory cytokine IL-6 and enhancement of anti-inflammatory cytokine IL-10 in the gut [1]. Additionally, IVIg therapy also led to increased expression of PPARγ, a ligand-activated transcription factor that mediates anti-inflammatory functions and resolution of inflammation, while TLR-4 expression, which mediates the inflammatory response, was reduced.

In general, sera from virtually all healthy adults contain anti-coronavirus antibodies [32]. Pyrc et al. showed that human sera from healthy adults inhibited HCoV-NL63 infection [10]. Additionally, they reported that IVIg can also neutralize HCoV-NL63 [10]. Boukhvalova et al. showed that, in contrast to commercially available polyclonal therapeutic IgG products, IVIg obtained from donors with high-titer antibodies against respiratory syncytial virus (RSV) have great potential to improve the outcome of RSV infection in immunocompromised subjects, not only by controlling viral replication but also by reducing damage to the lung parenchyma and epithelial airway lining [33,34].

Currently, all efforts to prevent the spread of COVID-19 so far have been inadequate. Immunotherapy with IgG can be employed to neutralize the virus causing COVID-19. The efficiency of IgG would be better if these immune IgG antibodies were collected from patients recovered from COVID-19 in the same city, or the surrounding area, as these donor subjects have naturally been confronted with the virus.

Immune IgG collected in Europe or the USA may be different from that collected in China as lifestyle, diet, and the environment play an important role in the development of specific antibodies against the virus. Recently, researchers at the Sacco University Hospital in Milan, Italy, have announced that they have isolated a new strain of coronavirus from an Italian patient that showed genetic differences when compared to the original strain isolated in China.

The idea is to treat infected patients with immune IgG collected from the same city in order to increase the chance of neutralizing the virus. Different procedures may be used to remove or inactivate any possible pathogens from the plasma of recovered coronavirus patient derived immune IgG, including solvent/detergent, 60 °C heat treatment, and nanofiltration (20 nm) [35,36,37,38]. Terpstra et al. showed that a 15 nm filtration step, combined with pepsin, and solvent-detergent treatment contribute to virus-elimination from liquid intravenous immunoglobulin [38].

Overall, immunotherapy with immune IgG combined with antiviral drugs could provide alternative treatment against COVID-19. These immune IgG antibodies collected from the recovered patients will be specific against COVID-19 by boosting the immune response in newly infected patients. Although a vaccine for COVID-19 is currently not available, the combination of the immune IgG antibodies with antiviral drugs can offer short-term and medium-term solutions against COVID-19.
